# Open challenges in developing digital therapeutics in the United States

**DOI:** 10.1371/journal.pdig.0000008

**Published:** 2022-01-18

**Authors:** Brenda Y. Miao, Douglas Arneson, Michelle Wang, Atul J. Butte

**Affiliations:** 1 Bakar Computational Health Sciences Institute, University of California, San Francisco, San Francisco, California; 2 Center for Data-Driven Insights and Innovation, University of California Health, Oakland, California; Massachusetts Institute of Technology, UNITED STATES

## Introduction

Digital therapeutics (DTx) are a rapidly emerging therapeutic modality that use evidence-based software to prevent, manage, or treat disease. In the United States, these new digital health tools fall under the FDA’s software-as-a-medical-device (SaMD) category and are subject to regulatory approval, much like conventional medical devices or therapeutic drugs [1]. The ability of DTx to deliver continuous and personalized care make them an attractive approach for chronic disease management, and they can be either used as standalone therapies or in combination with other treatments. Because DTx are typically administered through consumer devices, such as smartphones or tablets, they also have the potential to bridge significant care gaps created by shortages of specialized health providers and geographic barriers in accessing physical healthcare systems [2], but could also face other obstacles, such as the digital divide [3].

The COVID-19 pandemic accelerated the adoption of these digital solutions, especially with relaxed FDA guidelines allowing temporary approval of DTx offering psychiatric treatment for the duration of this public health emergency [4]. At least 63 approved DTx are now documented in the openFDA device databases, with 30 of these SaMDs approved after 2017 [5] (Table 1). The majority of these DTx (54/63) were approved through the 510(K) pathway with demonstrated substantial equivalence, three were approved as de novo applicants, and six SaMDs required premarket approval (PMA). Breaking down these devices by medical specialty, the most common categories for approved products were cardiovascular and anesthesiology, with 24 and 11 SaMDs respectively [5].

**Table 1:** Number of digital therapeutics extracted from openFDA device databases as of November 1, 2021, categorized by medical specialty (as reported by the FDA) and regulatory approval pathways.

		Approval type		
Medical Specialty	Approvals	510K	De Novo	PMA
**Cardiovascular**	24	24	-	-
**Anesthesiology**	11	11	-	-
**Unknown**	7	1	-	6
**Clinical Chemistry**	5	5	-	-
**General Hospital**	4	4	-	-
**Neurology**	3	2	1	-
**Ear, Nose, Throat**	2	2	-	-
**Gastroenterology, Urology**	2	1	1	-
**Obstetrics/Gynecology**	2	1	1	-
**Physical Medicine**	2	2	-	-
**General, Plastic Surgery**	1	1	-	-
**Total**	**63**	**54**	**3**	**6**

Funding for digital health companies, including the development of DTx, has grown exponentially in the past year, totaling $14.7 billion in just the first quarter of 2021 and already surpassing annual investments in 2020 ($14.6B) and 2019 ($7.7B) [6]. Both Hinge Health and MindMaze, which focus on chronic musculoskeletal conditions and substance abuse disorder, respectively, have raised over $100 million each in 2021 [7]. Although the financial backing for DTx supports their potential in providing healthcare at scale, many regulatory, commercial, and technical barriers remain in the adoption of these interventions into routine clinical care.

### Open challenges

Unlike previous therapeutics, DTx support rapid updates to their software to align with changes to best-practice clinical care guidelines or use adaptive algorithms that can improve over time as they encounter new data. This makes their use and predictability towards outcomes more challenging, and there is a pressing need for new regulatory frameworks in this field. To improve their ability to evaluate these increasingly complex DTx, the FDA launched a Software Precertification Pilot Program in 2017 [8]. Nine DTx companies selected for the program will help inform the development of a Total Product Lifecycle (TPLC) regulatory framework that would allow companies meeting precertification (“Excellence Appraisal”) criteria to market lower-risk devices with a Streamlined Review process followed by Real-World Performance monitoring. While the TPLC helps clarify the regulatory landscape for iterative DTx development, much work remains to ensure that this model is effective at producing safe and reliable DTx. As of the most recent update in September 2020, the FDA has drafted their metrics for Excellence Appraisal and an overview of the Streamlined Review process, and have begun exploring methods for collecting Real-World Performance data. This presents a significant challenge as collecting high quality data and modeling patient outcomes are non-trivial tasks, and are active areas of research in medical informatics [9]. For example, it is not clear whether medical orders for DTx are captured consistently in electronic medical record systems.

Reimbursement policies for DTx also remain under development as SaMDs are still being evaluated for treatment efficacy and cost-effectiveness. US-based pharmacy benefit managers Express Scripts and CVS Health both released digital health formularies in 2019 to identify recommended digital health solutions for insurers and physicians [10,11], but these only covered a limited number of medical specialties and many of the solutions are geared towards non-DTx mobile health apps that do not require FDA approval based on the agency’s “enforcement discretion” policies [8]. The Centers for Medicare and Medicaid Services (CMS) has also provided limited guidance, publishing some reimbursement codes to cover collaborative care models involving both clinical care and app usage, but not yet issuing codes for the use of DTx as a standalone treatment. SaMDs also do not fall under a particular Medicare benefit category, so breakthrough DTx are currently not eligible for coverage under new Medicare Coverage of Innovative Technology policies, further restricting their access to patient populations [12]. Finally, another major challenge with DTx reimbursement is capturing the value of the device itself, as well as the time that physicians spend monitoring patients and reviewing data from the apps.

As with any device storing and transmitting patient data, privacy and security are critical for DTx, especially those with frequent software updates or connections to other devices or electronic medical record systems. The FDA requires all medical devices companies to identify and mitigate cybersecurity risks as part of premarket submissions, but best-practice standards for data management and privacy are constantly evolving [13]. Another technical challenge, particularly for artificial intelligence and machine learning (AI/ML)-based DTx, is evaluating the safety and efficacy of algorithms in diverse patient populations. For example, training datasets may not always capture social determinants of health or other biases that affect the performance of healthcare algorithms [14]. Methods to recognize and remove such biases are paramount to ensuring the delivery of equitable healthcare, and device labeling requirements that clarify how these models were trained, or how they derived their outputs, may help promote transparency in AI/ML-based DTx [15].

## Conclusion

Addressing these regulatory, economic, and technical challenges will be crucial in bringing and successfully scaling DTx in the healthcare marketplace, and in fostering a digital health ecosystem that promotes patient access to SaMDs. With almost 150 FDA-regulated DTx expecting clinical trial results by 2022 [16], additional formularies and infrastructure around digital health education for patients and providers will help clarify questions on how to evaluate, prescribe, and effectively engage with DTx. Overcoming barriers around the development and deployment of DTx can significantly influence clinical care, and provide patients with novel therapeutic options in areas of unmet medical needs.
